# Cytokines, chemokines and antibodies against histone-3/4 citrullinated peptides in rheumatoid arthritis patients with pulmonary fibrosis

**DOI:** 10.1186/s13075-025-03603-x

**Published:** 2025-07-30

**Authors:** Linda Johansson, Federico Pratesi, Fosca Errante, Lorenzo Pacini, Paola Migliorini, Solbritt Rantapää-Dahlqvist

**Affiliations:** 1 Department of Public Health and Clinical Medicine, Umeå University, Umeå, SE-90185 Sweden; 2 Department of Translational Research and New Technologies in Medicine and Surgery, University of Pisa, Pisa, Italy; 3 PeptiLab, Interdepartment Research Unit of Peptide and Protein Chemistry and Biology, University of Florence, Florence, Italy; 4 Department of Clinical and Experimental Medicine, University of Pisa, Pisa, Italy

**Keywords:** Rheumatoid arthritis, Pulmonary fibrosis, Antibodies against citrullinated peptides, Histones, Cytokines, Chemokines

## Abstract

**Objective:**

Rheumatoid arthritis (RA) associated interstitial lung disease (ILD) is the most common pulmonary manifestations of RA, with a progressive course and a poor survival. An early detection and better treatment is essential to improve outcome. We evaluated 16 analytes that could be relevant for the development of RA ILD.

**Method:**

In an inception cohort of 1118 early RA patients, pulmonary fibrosis (PF) were identified in 60 patients after a mean follow-up of 5.3 years using high resolution computer tomography (HRCT). As controls, 124 early RA patients without PF and 94 matched population controls without known rheumatic disease were studied. Analysis of antibodies against histones 3 and 4 derived citrullinated peptides (CitH3/H4), and cytokines/chemokines levels were performed in plasma samples collected at RA diagnosis using in-house ELISA and Luminex analysis.

**Result:**

Anti-CitH3(114–135) antibodies were the only antibody with increased frequency and levels in patients with PF versus without PF. The highest OR for PF development were found when combining positivity for anti-CitH3(114–135) and -CitH4(31–50) antibodies, OR 2.26. Levels of IL1α, IL1ß, TNFα, VEGFA and MIPα remained significantly elevated in patients with PF compared without PF, after adjustments and Bonferroni corrections. Several of the cytokines/chemokines correlated significantly with the histone antibodies in patients without PF. Partial least squares discriminant analysis including antibodies against citrullinated histon peptides and cytokines/chemokines identified significantly in PF in non-smokers.

**Conclusion:**

Antibodies against CitH3 peptides and several of the analysed cytokines/chemokines in samples collected at diagnosis were associated with subsequent delevopment of PF in patients with RA.

**Supplementary Information:**

The online version contains supplementary material available at 10.1186/s13075-025-03603-x.

## Introduction

Rheumatoid arthritis (RA) associated interstitial lung disease (ILD) is the most common pulmonary manifestations of RA, with a progressive course and a poor survival [1]. The lungs have been suggested to serve as a potential site for RA disease initiation, and an increased amount of citrullinated peptides have been found in the tissue of the lungs of smokers [2, 3]. Smoking, in the presence of HLA-DRB1 shared epitope alleles, has been associated with an increased risk of developing anti-citrullinated protein antibody (ACPA) positive RA [4]. Infections and inflammation have been found to increase the citrullination of proteins in patients with RA and ILD [5, 6]. A broader ACPA repertoire found in RA patients with ILD have suggested a role for ACPA in the pathogenesis of lung disease [7].

Neutrophil extracellular traps (NETs) serve as a part of the innate immunity host defense against infections and represent as a source of citrullinated histones [8, 9], suggesting NETs playing a key role in the initiation and development of autoimmune disease and ACPA. Elevated levels of autoantibodies recognizing citrullinated peptides derived from histones released during NETosis, namely histone 3 (H3) and histone 4 (H4), have been observed before and after symptom onset of RA [8, 10, 11]. Moreover, histones released from NETs have been found to have the potential to damage alveolar epithelial cells, which in turn could cause a release of a large number of pro-inflammatory cytokines and chemokines. Among them, interleukin (IL)-6, IL8, IL1α, IL1β, and tumor necrosis factor-alpha (TNFα) that are released initiate subsequent inflammatory immune repair that may trigger fibrosis [10].

In this study of an inception cohort of RA patients, we investigate the association between antibodies against citrullinated peptides against histone H3- and H4- derived, and levels of plasma cytokines/chemokines to evaluate their potential role in the subsequent development of pulmonary fibrosis (PF).

## Materials and methods

### Study population

A case-control study was conducted within the cohort of early RA (eRA) patients participating in a larger study (*n* = 1118) performed to evaluate development of pulmonary fibrosis (PF) [11]. The patients with eRA (symptoms < 12 months) [12] were consecutively included between 1 January 1996 until 31 December 2016. Radiographic examinations were obtained of the lungs as part of clinical routine performed in 95% of the early RA patients. Therafter further radiographic examinations were performed using. High resolution computer tomography (HRCT) in the presence of any findings on plain X-rays or symptoms or signs of clinically suspect pulmonary involvement or other clinical indication of ILD [13]. The patients were followed clinically after inclusion every 6 months for the first 2 years and thereafter at clinical visits depending on the disease activity. Further radiographic examinations of the lungs were performed before biological treatment was started. The HRCT data spanning 20-year period were collected and analysed, focusing the assessment of pulmonary manifestations on pulmonary fibrosis (PF) including honeycombing, reticular patterns and/or traction bronchiectasis with ground-glass [14]. No additional diagnostic assessments were conducted to differentiate and isolate the manifestations of interstitial lung disease (ILD).

From the eRA cohort, PF was diagnosed in 60 patients (mean (SD) age 64.9 (10.3) years, 61.7% women) and 124 patients without PF from the same cohort (65.0 (9.8) years, 58.9% women) were included as RA controls in the study (Supplementary Table).

Clinical data e.g. DAS28 and pharmacological treatment were registered systematically as previous reported [13]. A total of 94 population controls without RA was extracted from the medical biobank of northern Sweden (NSHDS) with plasma samples available and were matched upon sex and age for the cases (65.0 (9.8) years, 58.9% women) (Supplementary Table). Smoking habits were registered as being a smoker, e.g. ever smoker, or non-smoker. All analyses of autoantibodies, cytokines and chemokines were conducted on plasma samples collected at the time of diagnosis of RA and stored at -80 °C until the time of analysis. None of the RA patients were on treatment with DMARDs or corticosteroids at the time of diagnosis of RA and blood sampling. The STROBE case-control reporting guidelines have been used [15].

### Peptide synthesis and analyses of antibodies against citrullinated H3 and H4 peptides

Linear peptides corresponding to the human histone H3 sequences H _31−20_ (Cit 2, 8, 17) and H3_114 − 135_ (Cit 116, 128, 129, 131, 134) and to citrullinated H4 _14−34_ (Cit 17, 19, 23) and H4_31 − 51_ (Cit 35, 36, 39, 40, 45) were synthesised by PeptLab (University of Florence, Italy). Peptides were prepared on an induction heating-assisted (PurePep Chorus, Gyros Protein Technologies) synthesiser or on a microwave-assisted (Liberty Blue, CEM) synthesiser following the Fmoc/tBu solid-phase peptide strategy. Peptides were purified by high performance liquid chromatography (HPLC) (purity > 95%) and characterised by electrospray ionisation mass spectrometry (ESI-MS).

ELISA polystyrene plates (Nunc MaxiSorp F96; Nunc, Roskilde, Denmark) were coated with H3 or H4 derived peptides at 5 µg/ml in 50 mM sodium carbonate/bicarbonate buffer pH 9.6 and incubated overnight at 4 °C. Saturation was carried out with PBS containing 1% porcine gelatin (Sigma Aldrich) for 45 min at room temperature. Sera diluted 1: 200 in PBS, 0.5% porcine gelatin and 0.05% Tween-20 were incubated on the plates for 3 h at room temperature. After washings with PBS, 1% Tween-20 and PBS, horseradish peroxidase (HRP)-conjugated anti human IgG (Sigma) diluted 1:5.000 was added to the wells, and the plates were incubated for 2 h at room temperature. After washings as above described, plates were incubated with tetra methyl benzidine for 15 min and H_2_SO_4_ 1 N solution was added before reading the absorbance at 450 nm. Results were expressed as percentage of an internal positive control run in each experiment.

### Analyses of anti-CCP2 antibodies and rheumatoid factor

The presence of anti-citrullinated protein antibodies (anti-CCP2) was determined by ELISA, utilizing a cut off of 25 AU/ml in accordance with the manufacturer’s protocol (EuroDiagnostica, Malmö, Sweden). Rheumatoid factor (RF) was determined by EliA assay using the Phadia 2500-system (Phadia GmbH, Freiburg, Germany)(cut-off at 20 AU/mL).

### Analyses of cytokines/chemokines

Cytokines and chemokines IL1α and β, TNFα, IL4, IL6, IL8, IL13, macrophage inflammatory protein (MIP)α and β, platelet-derived growth factor (PDGF)-AA/BB, Monocyte Chemoattractant Protein-1 (MCP1), vascular endothelial growth factor A (VEGFA)) were analyzed using Luminex technology (HCYTA-60 K, Human, Plasma– Human cytokine/chemokine/GrowthFactor Panel A, Merck, Darmstadt, Germany).

### Statistics

The statistical analysis was performed using SPSS software (v. 29.0 IBM Corp, Armonk, NY, USA) and R version 4.4. Student’s t-test was used for comparisons between groups for continuous data and chi-square test was used for comparisons of frequencies. Logistic regressions were used for identification of possible predictors for PF e.g. antibodies against peptides from citrullinated histones and cytokines/chemokines. Correlations were calculated using Spearman correlation coefficient. Adjustment for sex, age and smoking were made when appropriate. Data from logistic regression analyses were presented as odds ratio (OR) with 95% confidence interval (CI). A *p*-value < 0.05 were considered significant. Bonferroni–Holmes correction was used to adjust for multiple testing. Receiver operation characteristics (ROC) curves were generated, cut-offs for specifying antibody positivity/negativity for anti-histone antibodies was determined by maximizing the Youden index for RA cases vs. controls with respect to antibody concentration, under the additional condition that specificity was set at least 0.94. Partial least squares discriminant analysis (PLS-DA) was used to evaluate and illustrate differences in antibodies against citrullinated peptides of histones and cytokines/chemokines in the RA patients with PF versus without PF. The performance of the models is evaluated using 10 5-fold cross-validations resulting in an average balanced error rate (BER). The significance of the computed BER is assessed using a permutation test based on 1000 permutations and a permutation p-value is computed (pBER).

### Ethics

The study adheres to the principles of the Declaration of Helsinki, and ethical approval was obtained from the Regional Ethics Committee at Umeå University, Sweden (Approval Numbers: Dnr 2017-432-32 M, 2019–02039, 2022-06100-01). All participating patients provided informed consent before participating in the study.

## Result

### Levels of anti citrullinated histone H3/H4 derived peptides antibodies in patients with early RA without and with PF, and controls

**Fig. 1:**
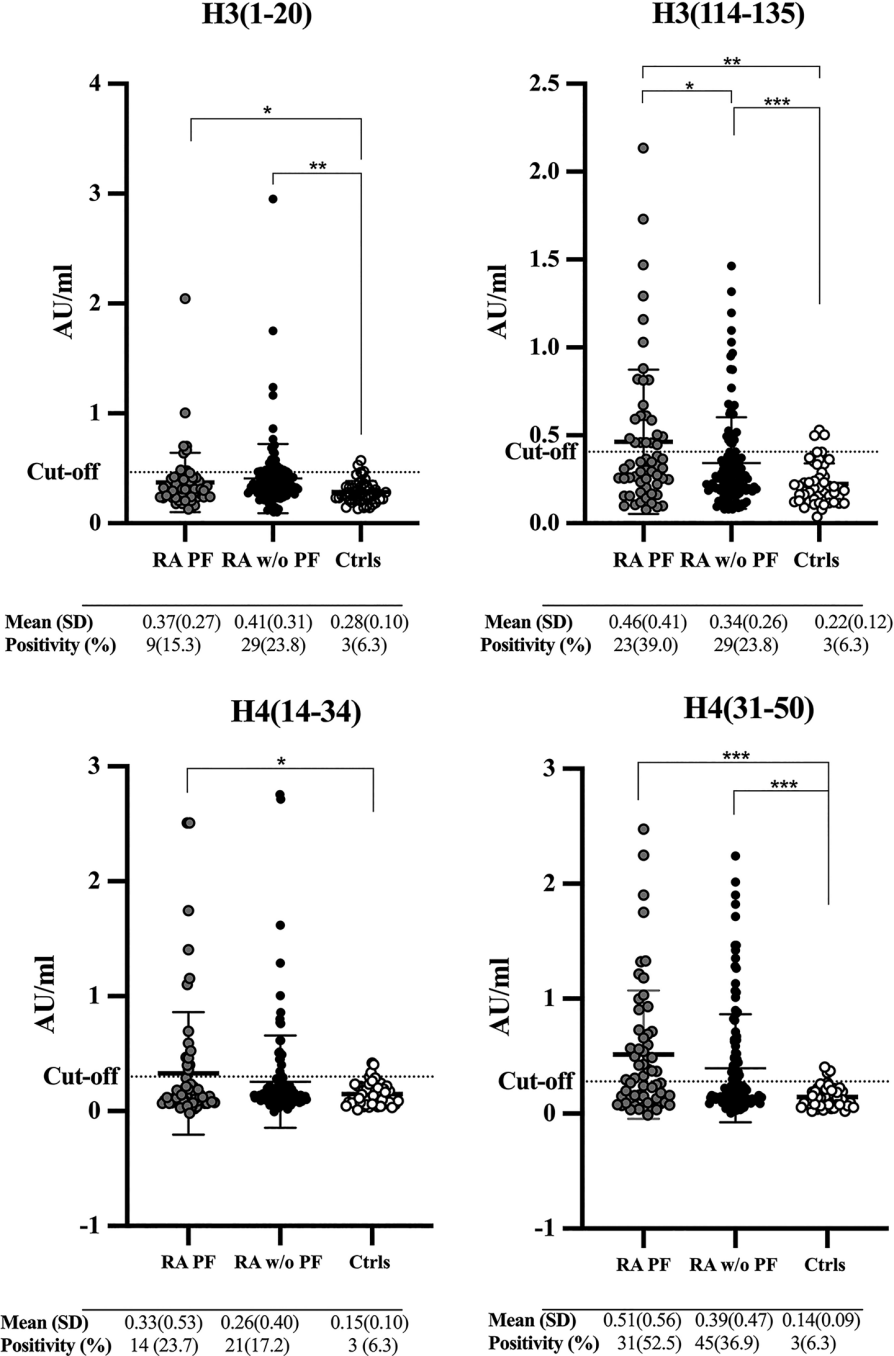
Concentrations of antibodies against citrullinated histone peptides from histone 3 and 4 (H3 and H4). Concentrations of antibodies against citrullinated histone peptides from histone 3 and 4 (H3 and H4) in rheumatoid arthritis patients with pulmonary fibrosis (RA PF, *n* = 59), without PF (RA w/o PF, *n* = 122), and in controls (Ctrls, *n* = 48). The dotted line indicated cut-off levels for antibody positivity. Mean (SD) presented below the X-axis and the number and percentage of cases above cut. *<0.05, **<0.01, ***<0.001. AU/ml = arbitrary units per milliliter

The concentrations of antibodies against histone citrullinated peptides were significantly elevated in RA patients irrespective of PF in comparison with controls, except for anti-CitH4(14–34) antibodies that was only increased in RA patients with PF (Fig. 1). RA patients with PF had also significantly higher concentration of anti-CitH3(114–135) antibodies, as compared with those RA patients without PF (*p* < 0.05) (Fig. 1).

### Frequency and combinations of positivity of anti citrullinated histone H3/H4 derived peptides antibodies in patients with RA with or without PF and controls

In RA patients with PF, antibodies against CitH4(31–50) were the most frequent, detected in 52.5%, followed by anti CitH3(114–135) in 39.0%. In RA patients without PF, lower antibody frequencies were observed, but again anti-CitH4(31–50) antibodies were the most frequent (36.9%), followed by anti-CitH3(1–20) and anti-CitH3(114–135) antibodies in 23.8%. The frequency of anti-CitH3(1–20), -CitH3(114–135) and -CitH4(31–50) were significantly increased in RA patients with PF vs. controls, independent of adjustment for sex, age and smoking (Table 1). Overall, 72.9% of RA patients with PF and 56.6% of RA patients without PF were positive for any of the antibodies, only 3.4% of RA patients with PF and 0.8% of the patients without PF were positive for all four antibodies *(*data not shown*).*

The highest OR for developing RA with PF vs. RA without PF was found for anti-H3(114–135) positive patients OR (95% CI) 2.01 (1.01, 4.01), *p* < 0.05 and the antibody combination that yielded the highest OR were H3(114–135) and H4(31–50) positive, OR (95%CI) 2.26 (1.16, 4.42), *p* < 0.05 (Table 1).

The association of CitH3 (114–135) antibody with pulmonary fibrosis was weakened when stratifying patients for RF positivity (OR = 2.25 (95%CI 0.82, 6.19) and anti-CCP2 antibody positivity (OR = 2.38 (95%CI 0.89, 6.40). However the presence of anti-CCP2 antibodies was associated with increased concentations of antiCit H4 (14–34) (0.12 AU/ml to 0.38 AU/ml, *p* = 0.025) and antiCit H4(31–50) (0.14 AU/ml to 0.61 AU/ml, *p* < 0.001) in patients with fibrosis.

**Table 1:** Frequency above cut-off at 93.7% specificity for anti-Cit H3(1–20), -H3(114–135), -H4(14–34) and H4(31–50) antibodies in rheumatoid arthritis (RA) patients vs. controls and odds ratio (OR) between RA patients with and without pulmonary fibrosis (PF) adjusted for. Sex, age and smoking

Antibodies	Antibody frequency n (%) in RA patients with PF (*n* = 59)	Antibody frequency n (%) in RA patients without PF (*n* = 122)	RA patients with PF vs. without PF OR (95% CI)
H3(1–20)	9 (15.3)*	29 (23.8)***	1.6 (0.67, 3.85)
H3(114–135)	23 (39.0)***	29 (23.8)	2.01 (1.01, 4.01)*
H4(14–34)	14 (23.7)	21 (17.2)	1.74 (0.80, 3.80)
H4(31–50)	31 (52.5)***	45 (36.9)***	1.24 (0.59, 2.61)
H3(1–20) + H3(114–135)	25 (42.4)**	47 (38.5)***	1.21 (0.63, 2.35)
H3(1–20) + H4(14–34)	20 (33.9)*	39 (32.0)**	1.28 (0.64, 2.57)
H3(1–20) + H4(31–50)	36 (61.0)***	58 (47.5)***	1.76 (0.89, 3.46)
H3(114–135) + H4(14–34)	31 (52.5)***	42 (34.4)**	2.24 (1.16, 4.33)*
H3(114–135) + H4(31–50)	39 (66.1)***	57 (46.7)***	2.26 (1.16, 4.42)*
H4(14–34) + H4(31–50)	35 (59.3)***	52 (42.6)***	1.92 (0.99, 3.70)*

*p < 0.05, **p < 0.01, ***p < 0.001, n = number, CI = confidence interval

### Levels of cytokines/chemokines in patients with early RA with or without PF and controls

**Fig. 2:**
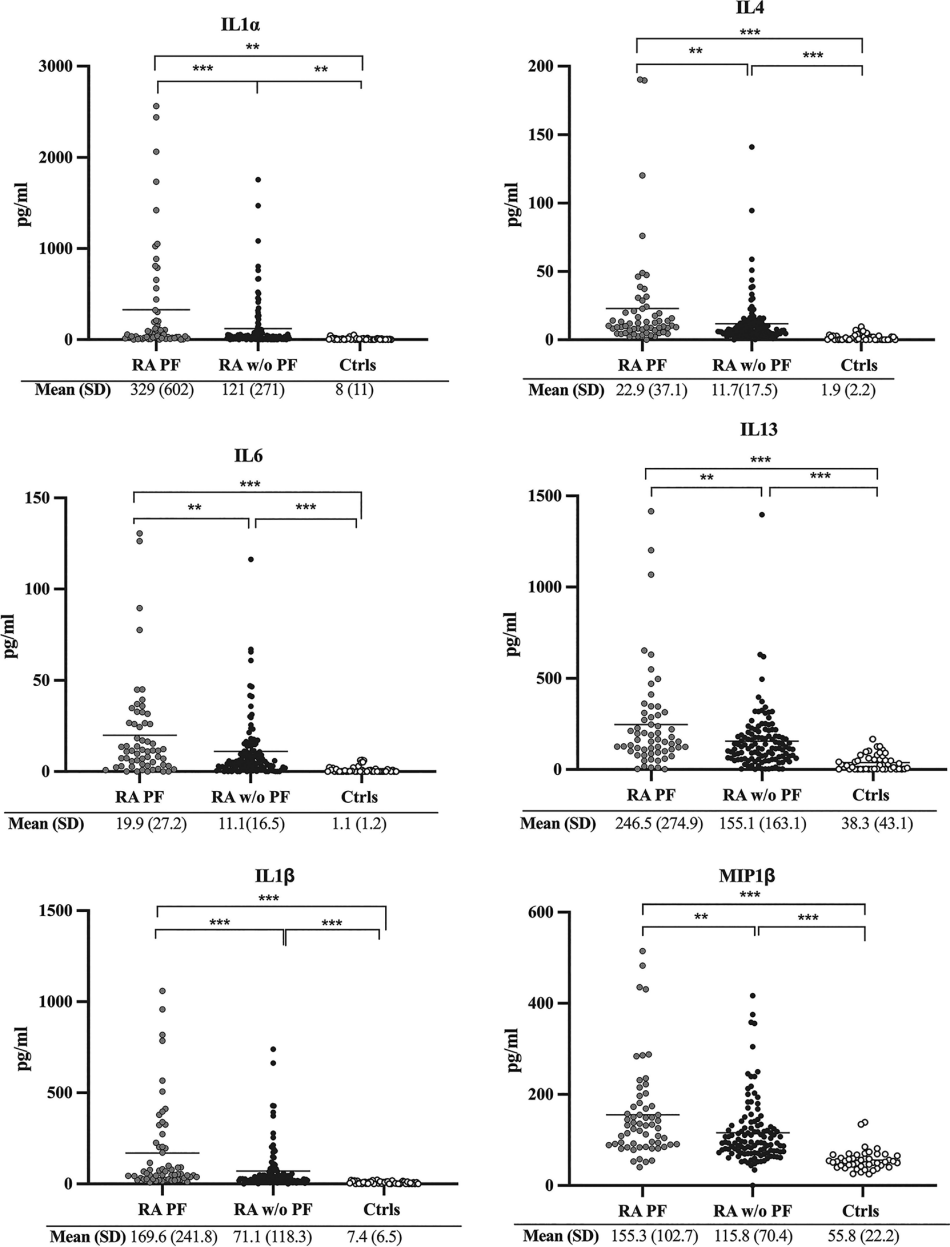

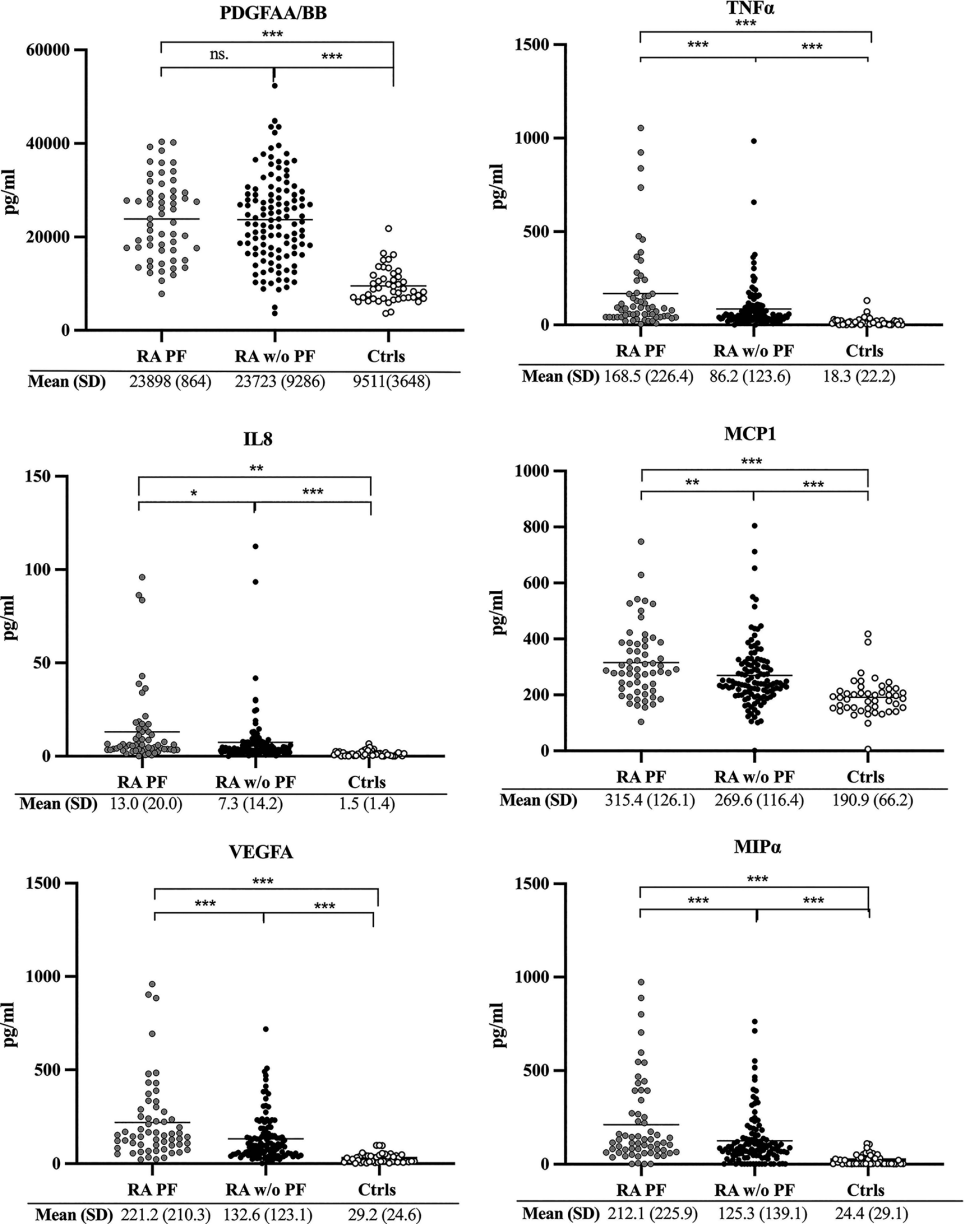
Concentrations of cytokines/chemokines in rheumatoid arthritis (RA) patients with and without pulmonary fibrosis (PF). Concentrations of cytokines/chemokines in rheumatoid arthritis (RA) patients with pulmonary fibrosis (PF) (*n* = 59) and without (w/o) PF (*n* = 118), and in controls (*n* = 46). Mean (SD) presented below the X-axis. *<0.05, **<0.01, ***<0.001, ns = non significant, (pg/ml) = picograms per milliliter

Significantly higher levels of IL1α, IL4, IL6 IL13, IL1ß, MIP ß, PDGF-AA/BB, TNFα, IL8, MCP1, VEGFA, MIPα were found in RA in comparison with controls, independent of PF status (Fig. 2). Furthermore, significantly elevated levels of IL1α, IL4, IL6, IL13, IL1ß, MIP ß, TNFα, IL8, MCP1, VEGFA, MIPα, except for PDGF-AA/BB were seen in PF patients compared with patients without PF (*p* < 0.01 − 0.001). These calculations were unadjusted (Fig. 2)*.* Only IL1α, IL1ß, TNFα, VEGFA and MIPα remain significantly elevated in RA-PF after Bonferroni-correction (data not shown*).*

The highest OR for developing PF in RA were found for IL4, OR (95%CI) 1.018 (1.003, 1.035), *p* < 0.05, adjusted for sex, age and smoking (Table 2). For RA versus controls IL8 had the highest OR; RA with PF OR (95%CI) 2.29 (1.55, 3.37), *p* < 0.001 and without PF OR (95% CI) 2.53 (1.74, 3.69), *p* < 0.001, adjusted for sex, age and smoking (Table 2). In further adjustment with disease acticity (DAS28), besides age, sex and smoking, the significant effects of IL8 and MCP1 was reduced, OR = 1.018 (95%CI 0.998, 1.038), *p* = 0.08 and 1.003 (1.000, 1.005), *p* = 0.055, respectively. All other cytokines/chemokines remained significantly associated with PF.

**Table 2:** The odds ratio (OR) of cytokines/chemokines in rheumatoid arthritis (RA) patients with and without pulmonary fibrosis (PF), and controls. OR adjusted for sex, age and smoking

Cytokines/chemokines	RA patients with PF vs. controls OR (95% CI)	RA patients without PF vs. controls OR (95% CI)	RA patients with PF vs. RA without PF OR (95% CI)
IL1α	1.08 (1.04, 1.13)***	1.07 (1.03, 1.11)***	1.001 (1.00, 1.002)**
IL4	1.84 (1.44, 2.36)***	1.81 (1.45, 2.26)***	1.018 (1.003, 1.035)*
IL6	1.65 (1.31, 2.07)***	1.67 (1.34, 2.10)***	1.020 (1.003, 1.036)
IL13	1.03 (1.02, 1.04)***	1.03 (1.02, 1.03)***	1.002 (1.001, 1.004)*
IL1β	1.40 (1.14, 1.69)***	1.20 (1.12, 1.29)***	1.004 (1.001, 1.006)**
MIP ß	1.06 (1.04, 1.09)***	1.07 (1.04, 1.09)***	1.006 (1.002, 1.010)**
PDGFAA/BB	1.00 (1.00, 1.00)***	1.00 (1.00, 1.00)***	**1.00 (1.00, 1.00)**
TNFα	1.06 (1.03, 1.10)***	1.06 (1.04, 1.09)***	1.003 (1.001, 1.005)**
IL8	2.29 (1.55, 3.37)***	2.53 (1.74, 3.69)***	1.021 (1.000, 1.042)
MCP1	1.09 (1.01, 1.03)***	1.01 (1.01, 1.02)***	1.003 (1.001, 1.006)*
VEGFA	1.06 (1.03, 1.08)***	1.05 (1.03, 1.07)***	1.004 (1.001, 1.006)***
MIPα	1.04 (1.02, 1.06)***	1.03 (1.02, 1.05)***	1.003 (1.001, 1.005)**

Corrected p-value, with Bonferroni *p < 0.05, **p < 0.01, ***p < 0.001, n = number, CI = confidence interval

### Levels of cytokines/chemokines in relationship with anti-citrullinated histone antibodies, in patients with RA with and without PF

**Fig. 3:**
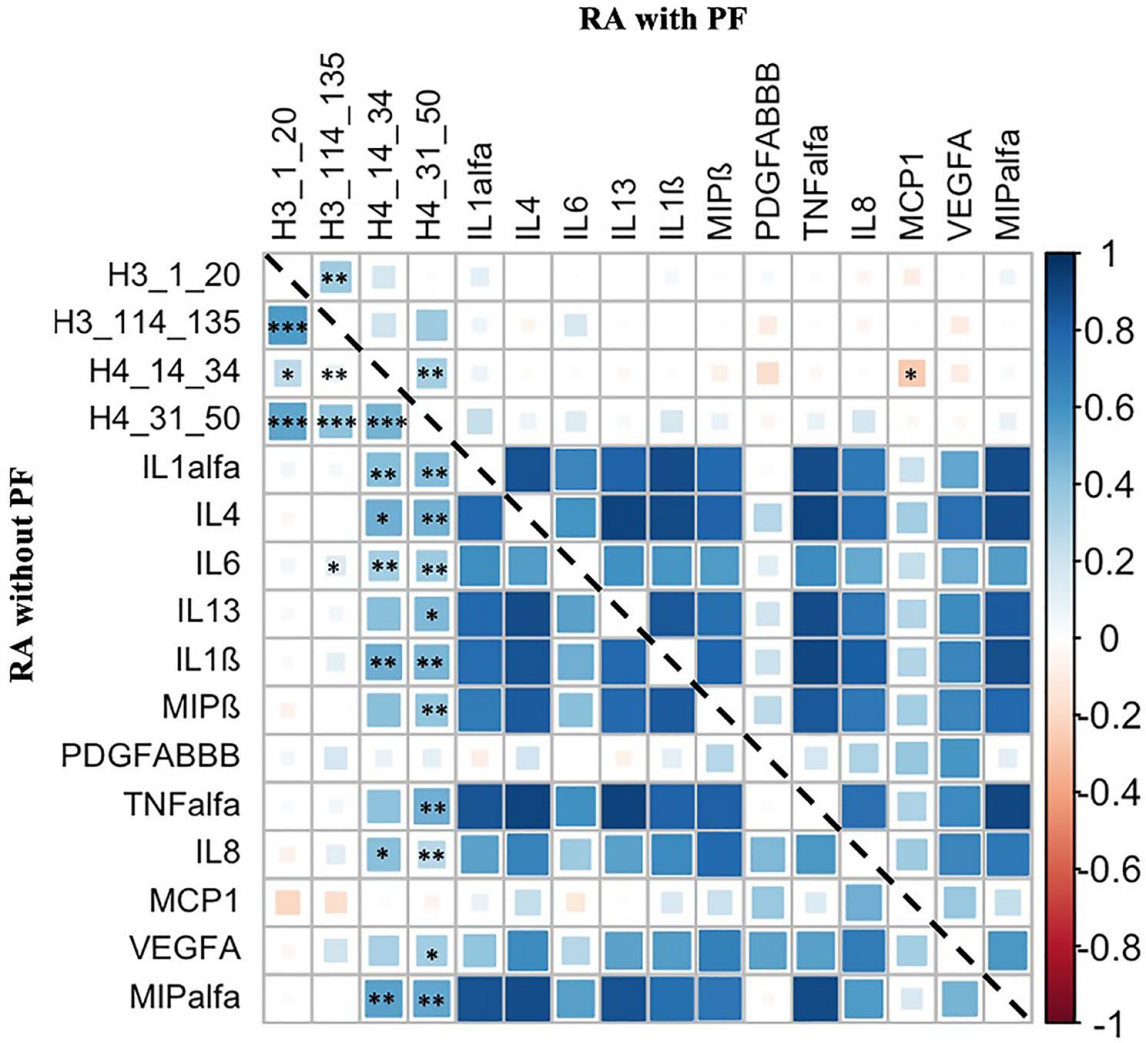
Correlation plot showing antibodies against citrullinated histone 3 and histone 4 peptides, and cytokines/chemokines. Correlation plot showing antibodies against citrullinated histone 3 and histone 4 peptides, and cytokines/chemokines calculated with Spearman correlation coefficient. The colour illustrates the strength of correlation, blue = positive correlation and red = negative correlation. Upper quadrant = patients with pulmonary fibrosis and lower quandrant = patients without pulmonary fibrosis. Only significant correlations between antibodies against citrullinated histones 3 and 4 peptides and cytokines/chemokines were illustrated. *p < 0.05, **p < 0.01, ***p < 0.001

In RA with PF (Fig. 3 - data above the dotted line), levels of anti-Cit H3(1–20) correlated significant with anti-Cit H3(114–135) antibodies (r_s_=0.37, *p* < 0.01). Anti-Cit H4(14–34) correlated with anti-Cit H4(31–50) antibodies (r_s_=0.35, *p* < 0.01). Among them, only anti-CitH4(14–34) antibodies showed a significant inverse correlation with cytokines/chemokins in PF, MCP-1, (r_s_= -0.265, *p* = 0.05).

In RA patients without PF (Fig. 3 - data below the dotted line*)*, several of the antibodies against histone 3 and 4 peptides correlated significantly with each other (Fig. 3). Futhermore, many of cytokines/chemokines did also correlate significantly with anti-CitH3(114–135), -CitH4(14–34) and -CitH4(31–50) antibodies. Anti-CitH3(114–135) antibodies correlated with IL6 and anti-CitH4(14–34) antibodies with IL1α, IL4, IL6, IL1ß, IL8 and MIPα. Anti-CitH4(31–50) antibodies correlated significantly with IL1α, IL4, IL6, IL13, IL1ß, MIPß, TNFα, IL8, VEGFA and MIPα (Fig. 3*).*

### Partial least squares discriminant analysis (PLS-DA)

**Fig. 4:**
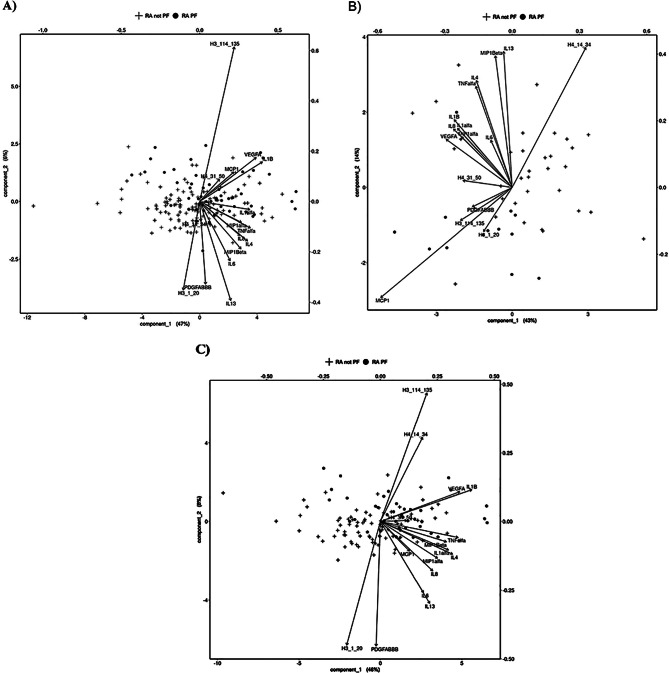
Partial least squares discriminant analysis (PLS-DA) for separation of the three groups. Partial least squares discriminant analysis (PLS-DA) for separation of the groups. (**A**) RA with PF and RA without PF, BER = 0.47, p.BER = 0.091, (**B**) non-smokers BER = 0.35 and p.BER = 0.023 and (**C**) separated for smokers, BER = 0.531, p.BER = 0.12. Biplot based on cytokines/chemokines levels and antbody against citrullinated histones peptides (H3/H4) positivity

Partial least squares discriminant analysis (PLS-DA) were performed to investigate the separation of the two groups, RA with PF and without PF using the levels of cytokines/chemokines and frequencies of antibodies against citrullinated peptides of histones. There was a weak separation between the RA patients with and without PF (BER = 0.47, p.BER = 0.091) Fig. 4A. When stratified on smoking habits, a significant separation between RA with and without PF was found based on antibodies against histones and cytokines/chemokines in non-smokers, BER = 0.35 and p.BER = 0.023 (Fig. 4B. In smokers this separation could not be seen, BER = 0.531, p.BER = 0.12 (Fig. 4C).

## Discussion

In this present study, investigating an inception cohort of patients with early RA we have identified antibodies against citrullinated peptides from H3 and several cytokines/chemokines as potential biomarkers for development of PF in RA. Previously, a peptide from citrullinated histone-4 from activated neutrophils has been shown to be targets for ACPA in sera from patients with RA [8] with increased antibody levels years before and after symptom onset of RA, compared with controls [8, 11]. In this study we could confirm our previous findings with increased antibody levels against peptides from citrullinated histone 3 and 4, in patients with RA compared with controls [8]. Among the anti-histones antibodies, anti-CitH3(114–135) antibodies were the only antibody with increased levels and antibody positivity in patients with PF as compared with patients without PF. The highest OR, 2.26, for PF development was found when combining positivity for anti-CitH3(114–135) and anti-CitH4(31–50) antibodies.

Previous studies have shown increased NETosis in patient with PF and RA [10, 16], suggesting a role of NETs to serve as a potential source of citrullinated histones playing a key role in the initiation and development of autoimmune disease and ACPA production. Furthermore, short telomeres were strongly associated with prevalent but not incident ILD among patients with RA. Histones have been found to play an important regulatory role in telomere length increasing the risk of PF, and RA-ILD [17, 18]. A previous study by Kronzer et al. showed an increased risk of RA-ILD in patients with antibodies against histone 2 A, while antibodies against histone 4 were inversely associated with RA-ILD [19]. In this present study, we did not find a relation between the immune response to H4 and PF in RA. However, multiple differences between the studies may explain these different results. The patient cohorts are not identical and we did not select RA patients affected by ILD but enrolled those affected by PF. Additionally, our analysis was limited to measure IgG antibodies against citrullinated peptides from H4, rather than antibodies targeting the entire H4 molecule.

Even if several cytokines/chemokines have through the years been suggested to regulate different inflammatory pathways in idiopathic pulmonary fibrosis (IPF) [10, 20], studies investigating inflammatory makers in RA-ILD are still limited. Thus, we analyzed a panel of cytokines and chemokines in our patients. Among them, IL1α, IL1ß, TNFα, VEGFA and MIPα remained significantly elevated in patients with PF versus those without PF, after adjustments for sex, age and smoking and multiple testing. A role of these cytokines and chemokines in lung fibrosis has been suggested in several studies in experimental models or in the human disease [21, 22]. IL6 differed significantly in RA with PF vs. those without, but the significance was lost when corrected for multiple testing. The levels of IL-6 correlated significantly with anti-CitH3(114–135) and -CitH4(31–50) antibodies in patients without PF. Previous studies in idiopathic pulmonary fibrosis (IPF), have shown increased levels of IL-6 in patients with acute exacerbation of IPF, compared with patients with stable IPF [21]. In mice, IL1β was crucial for intiating the inflammatory response and fibrosis progression through IL-1R1/MyD88 [22]. Both, IL1β and IL6 have been found to promote fibrosis via TGF-β and STAT3 signaling, respectively [23, 24]. Furthermore, IL-1α is secreted by alveolar epithelial during stress, promotes the formation of pro-inflammatory fibroblast leading to cytokine relase and promotion of PF [25]. TNFα have been shown to be secreted from lung fibroblast and indirectly promote fibrosis [26, 27]. Despite these findings, in RA the benefits and risk of treatment with TNFα inhibitors for ILD are still uncertain [28]. The angiogenic factor VEGFA have previously been shown to correlate with disease activity in RA and was increased in RA-ILD [29]. The pro-inflammatory chemokine MIPα is responsible for monocyte recruitment, and was upregulated in fibrotic lungs from mice, and BAL fluid from patient with IPF [30]. IL4 together with IL13 as part of type 2 inflammation have been suggested to play a role in IPF both in rodent experimental studies and clinical analyses [31, 32], which is of interest as we found IL-4 with the highest odds ratio in our RA-PF patients. In another study on 35 RA patients with ILD a number of cytokines/chemokines, some similar to those of our study, were significantly increased compared with RA without ILD, e.g., IL1α, IL6, IL8, IL18, IL23, MCP1 and MIP1ß In a Cox regression analyis, disease activity, high ACPA titre, IL18, MCP1 and SDF1α were associated with ILD [33]. Active disease has been identified as a risk factor for PF as well as presence of RF and/or ACPA as anti-CCP2 antibodies [4]. In another study on a larger cohort, we only found that RF was associated with PF, age at RA onset, RF positivity and methotrexate treatment at onset predicted significantly PF [13]. Concerning ACPA we have also found significant associations with several ACPA fine specificities and PF but not to anti-CCP2 positivity and level or RF positivity [35].

Analysing the relationship between cytokines/chemokines levels and anti peptide antibodies, we found many correlations but only in patients without PF. In RA PF patients, anti-CitH4(14–34) antibodies correlated negatively with MCP-1, (r_s_= -0.265, *p* = 0.05). The levels of IL-6 correlated significantly with anti-CitH3(114–135) and -CitH4(31–50) antibodies in patients without PF. The lack of correlations in RA-PF patients could result from low number of patients. We analyzed the overall contribution of anti peptide antibodies and cytokines/chemokines in distinguishing RA patients with or without PF by means of PLS. When the confounding effect of smoking was taken out, a significant separation between RA with and without PF was found based on antibodies against histones and cytokines/chemokines in non-smokers. Antibodies anti-CitH4(14–34) together with MCP1 emerged as the most evident variables in separating the two populations.

The strength of the current study is that the cohort of RA patients originated from a homogeneous population of northern Sweden, and were followed on a regular basis after disease onset. X-rays of the lungs were performed routinely at inclusion on all of patients, providing baseline information about the lungs. A limitation of the study is that the HRCT examinations were not performed on all included patients, only for those with abnormalities on the plain X-rays or with clinical symptoms or signs. Although, the HRCT examinations have been performed over a period of almost 20 years methodological improvements during this time could affect the results. Another limitation was that the number of patients with PF was rather low, which could have an impact on the statistical calculations of the relationships between cytokines/chemokines and antibodies against histones. The controls were identified from a population-based biobank from northern Sweden, except for RA, information about any comorbidities among the controls are lacking. The prevalence of IPF in a study from a neighbor country, Finland, was 36.0 per 100 000 in 2021, suggesting the prevalence among the controls should be low [36]. Furthermore, another limitation was that not all of the controls hade enough plasma samples to be analysed for both antibodies and cytokines/chemokines.

Even taking into account these limits, we can conclude that this study allows to identify a subset of anti histone ACPA and a group of cytokines and chemokines associated with the subsequent development of PF in an inception cohort of RA.

## Electronic supplementary material

Below is the link to the electronic supplementary material.

Supplementary Material 1

## Data Availability

No datasets were generated or analysed during the current study.
