# Supplementary material for: Cytokines, chemokines and antibodies against histone-3/4 citrullinated peptides in rheumatoid arthritis patients with pulmonary fibrosis
Source: Arthritis Res Ther. 2025 Jul 30;27:160. doi: 10.1186/s13075-025-03603-x (PMC12308956; doi:10.1186/s13075-025-03603-x)
Supplement: Supplementary file 1 — Supplementary Material 1 [file 13075_2025_3603_MOESM1_ESM.docx]

**Supplementary Table.** Demographic data and treatment at inclusion on rheumatoid arthritis (RA) patients with and without pulmonary fibrosis (PF) and controls, analysed for antibodies against citrullinated histone (H3/H4) derived peptides and/or cytokines/chemokines.

|  | **With PF**  **N=60** | | **Without PF**  **N=124** | | **Controls**  **n=94** | |
| --- | --- | --- | --- | --- | --- | --- |
|  | *Antibodies^a^*  *N=59* | *Cytokines^b^*  *N=59* | *Antibodies^a^*  *N=122* | *Cytokines^b^*  *N=118* | *Antibodies^a^*  *N=48* | *Cytokines^b^*  *N=46* |
| **Age at sampling, mean (SD)** | 64.7(10.4)***^2^ | 64.7(9.8)***^2^ | 65.1(9.9)***^3^ | 64.8(10.4)*^3^ | 54.7(9.9) | 59.4(9.0) |
| **Age at diagnose of PF, mean (SD) age** | 73.8 (9.9) | 73.8 (9.9) | - | - | - | - |
| **Women, n (%)** | 36/59(61.0) | 37/59(62.7) | 71/122(58.2) | 69/118(58.5) | 30/48(62.5) | 26/46(56.5) |
| **Follow-up after RA diagnosis, mean (SD),years** | 8.7 (4.8) | 8.7 (4.8) | 10.9 (4.9) | 10.9 (4.9) | - | - |
| **Ever smoker, n (%)** | 42/57(73.2)***^2^ | 42/57(73.7)*^2^ | 87/122(71.3)***^3^ | 84/118(71.2)*^3^ | 20/48(42.6) | 23/46(50) |
| **Anti-CCP2 positive, n (%)** | 47/59(79.7)***^2^*^1^ | 47/59(79.7)***^2^ | 75/122(61.5)***^3^ | 73/118(61.9)***^3^ | 2/48(4.2) | 5/46(10.9) |
| **RF positive, n(%)** | 52/59(88.1)**^1^ | 52/59(88.1)*^1^ | 85/122(69.7) | 81/118(68.6) | - | - |
| **DAS28-AUC**  **mean (SD)** | 90.3 (18.8) | 90.3 (18.8) | 85.7 (20.1) | 85.7 (20.1) | - | - |
| **DAS28 baseline, mean (SD)** | 4.8 (1.4) | 4.8 (1.4) | 4.8 (1.4) | 4.8 (1.4) | - | - |
| **CRP**  **baseline, mg/L**  **Mean (SD)** | 21.6 (18.5) | 21.6 (18.5) | 24.6 (29.7) | 24.6 (29.7) | - | - |
| **Methotrexate at baseline, n (%)** | 37/56(66.1)*^1^ | 39(66.1)*^1^ | 83/93(89.2) | 92/115(80) | - | - |
| **Prednisolone at baseline, n (%)** | 36/59(61.0) | 36/59(61.0) | 82/121(67.8) | 80/116(69.0) | - | - |

^a^individuals with analysis of antibodies citrullinated histone peptides ^b^individuals with analysis of cytokines/chemokines, PF – Pulmonary fibrosis, SD – Standard deviation, n – number, anti-CCP2 – antibodies against cyclic citrullinated protien 2, RF – rheumatoid factor

^1^between RA patients with PF vs. without PF ^2^between RA patients with PF vs. controls ^3^between RA patients without PF vs. controls *p<0.05, **p<0.01, ***p<0.001
